# Insulin-Like Growth Factor-2 (IGF-2) Does Not Improve Memory in the Chronic Stage of Traumatic Brain Injury in Rodents

**DOI:** 10.1089/neur.2021.0031

**Published:** 2021-10-20

**Authors:** John B. Redell, Mark E. Maynard, Kimberly N. Hood, Anthony N. Moore, Jing Zhao, Pramod K. Dash

**Affiliations:** Department of Neurobiology and Anatomy, The University of Texas McGovern Medical School, Houston, Texas, USA.

**Keywords:** cortical impact injury, fear extinction, novel object recognition, water maze

## Abstract

Persistent cognitive impairment(s) can be a significant consequence of traumatic brain injury (TBI) and can markedly compromise quality of life. Unfortunately, identifying effective treatments to alleviate memory impairments in the chronic stage of TBI has proven elusive. Several studies have demonstrated that insulin-like growth factor-2 (IGF-2) can enhance memory in both normal animals and in experimental models of disease. In this study, we questioned whether IGF-2, when administered before learning, could enhance memory performance in the chronic stage of TBI. Male C57BL/6 mice (*n* = 7 per group) were injured using an electronic cortical impact injury device. Four months later, mice were tested for their cognitive performance in the fear memory extinction, novel object recognition (NOR), and Morris water maze tasks. Twenty minutes before each day of training, mice received a subcutaneous injection of either 30 μg/kg of IGF-2 or an equal volume of vehicle. Memory testing was carried out 24 h after training in the absence of the drug. Uninjured sham animals treated with IGF-2 (or vehicle) were trained and tested in the fear memory extinction task as a positive control. Our data show that although IGF-2 (30 μg/kg) improved memory extinction in uninjured mice, it was ineffective at improving fear memory extinction in the chronic stage of TBI. Similarly, IGF-2 administration to chronically injured animals did not improve TBI-related deficits in either NOR or spatial memory. Our results indicate that IGF-2, administered in the chronic stage of injury, is ineffective at enhancing memory performance and therefore may not be a beneficial treatment option for lingering cognitive impairments after a TBI.

## Introduction

Traumatic brain injury (TBI) is a major health concern for persons of all ages. One of the persistent consequences of TBI is memory dysfunction. Depending on the severity of the TBI, memory impairments can last for days to months to years and, in some cases, may last a lifetime. The hippocampus is particularly vulnerable to TBI, and damage to this limbic structure has been demonstrated to impair declarative memory in humans and navigation- and context-specific memories in rodents.^1–4^ Unfortunately, effective treatments to ameliorate memory impairments in persons living with the long-term consequences of TBI are currently not available.

Both clinical and experimental studies have demonstrated that insulin, as well as insulin-like growth factors-1 and -2 (IGF-1 and IGF-2, respectively), can improve cognitive function in normal animals, as well as in animal models of disease such as Alzheimer's disease.^5–10^ Insulin, IGF-1, and IGF-2 readily cross the blood–brain barrier and can act upon central nervous system cells expressing their respective receptors.^11,12^ A study by Stern and colleagues individually injected each of these proteins directly into either the ventricle, amygdala, or dorsal hippocampus to assess the efficacy of each factor to enhance memory.^13^ These investigators observed that direct injection of insulin, IGF-1, or IGF-2 into the amygdala did not have any effect on amygdala-dependent fear memory. In contrast, direct injection of IGF-2 into the hippocampus showed a robust enhancement of hippocampus-dependent long-term memory formation, whereas insulin injection produced only a transient improvement, and IGF-1 injection had no effect. A series of key studies by the same group has clearly established IGF-2 to be a potent cognitive enhancer that can effectively improve memory in normal animals after either direct injection into brain structures or systemic administration.^6,14,15^

In the present study, we examined whether systemic administration of IGF-2 could enhance memory when administered to mice 4 months after a TBI. This chronic time frame was chosen to allow time for acute injury-induced pathologies to stabilize and represents a time frame during which we have previously observed lingering cognitive dysfunction. To establish that the experimental paradigm was effective, we first demonstrated that systemic IGF-2 (30 μg/kg, subcutaneously [s.c.]) was able to enhance extinction of a conditioned fear memory in uninjured animals, as has been shown by others.^13–15^ Next, three different memory tasks (extinction of context fear, novel object recognition [NOR], and Morris water maze [MWM]) were used to test the efficacy of IGF-2 treatment to improve memory performance in mice 4 months after a moderate-severe controlled cortical impact (CCI) injury. Our results show that systemic IGF-2 administration failed to improved memory in any of the tasks assessed, suggesting that it may not be a viable treatment option for alleviating memory dysfunction in the chronic stages of TBI.

## Methods

### Animals

Twenty-eight adult male C57BL/6 mice (*n* = 7 per treatment group; The Jackson Laboratory, Bar Harbor, ME) were single-housed on a 12-h light/dark cycle, with *ad libitum* access to food and water. Experiments were performed during the animal's light cycle. All experimental procedures were conducted in accordance with the Guide for the Care and Use of Laboratory Animals of the National Institutes of Health and were approved by the Animal Welfare Committee of UTHealth (University of Texas Health Science Center at Houston, Houston, TX).

### Controlled cortical impact injury

An electromagnetic CCI device (Leica Biosystems, Buffalo Grove, IL) was used to cause moderate-severe brain injury in mice, as has been previously described.^16–18^ Briefly, male C57BL/6 mice (∼30 g) were deeply anesthetized in an induction chamber by exposure to 5% isoflurane in a 1:1 O_2_/air mixture for 4 min. Animals were mounted on a stereotaxic frame using both incisor and ear bars, and anesthesia maintained throughout the surgery and injury with 2.5% isoflurane in a 1:1 mixture of O_2_/air administered by face mask. A 4-mm-diameter craniectomy was made over the parietal cortex, and the injury—consisting of a single impact at 3 m/sec, 0.5 mm deformation, and 200 msec dwell time—was administered to the right parietal cortex using a 3-mm flat-bottomed impactor tip. After injury, animals were allowed to recover in a warmed chamber before being returned to their home cages. Beginning 4 months after the injury, animals were randomly divided into either IGF-2 or vehicle groups (*N* = 7 per group) for behavioral testing.

### Drug preparation and administration

Recombinant mouse IGF-2 (R&D Systems, Minneapolis, MN) was dissolved in 0.1% bovine serum albumin/phosphate-buffered saline (0.1% BSA-PBS) and administered s.c. at a dose of 30 μg/kg 20 min before training for each of the behavioral assessments. Stern and colleagues previously demonstrated, using a dose-response experiment, that a 30-μg/kg (s.c.) dose of IGF-2 administered 20 min before contextual fear conditioning (CFC) provided the strongest enhancement of fear memory.^15^ This dose has been also shown to improve memory performance in a variety of animal models and behavioral tasks.^5,15,19–23^ Vehicle-treated groups received an equivalent volume of 0.1% BSA-PBS.

### Contextual fear conditioning and extinction

A one-trial CFC procedure was used to investigate the effect of IGF-2 on freezing behavior and extinction of fear memory in both uninjured and brain-injured mice. The training chamber included only visual cues. No auditory stimulus (i.e., tone) was used in training. Animals were injected with either IGF-2 (30 μg/kg s.c.) or vehicle 20 min before training. Animals were then placed in the training chamber and allowed to explore freely within the context for 2 min before receiving a brief, mild foot shock (2.0 sec, 0.7 mA). After the shock, mice remained in the context for an additional minute before being removed and returned to their home cage. Twenty-four hours later, animals were placed back in the conditioning chamber for 3 min (in the absence of a foot shock reminder), and fear memory was measured by monitoring freezing behavior using Ethovision software (Noldus, Wageningen, the Netherlands). Extinction of fear memory was carried out by exposing mice to the training chamber for 10 min per day for 6 consecutive days. Twenty minutes before each daily extinction trial, animals were injected with either IGF-2 (30 μg/kg s.c.) or vehicle. Freezing behavior was measured during the first 2 min of each extinction trial and used as a measure of memory for the previous day's extinction training.

### Novel objection recognition

Brain-injured animals were placed in an opaque plastic box (L × W × H; 42 × 20 × 20 cm) inside of a sound-attenuated chamber and allowed to habituate for two 10-min periods per day for 2 days. On day 3, animals were administered either IGF-2 (30 μg/kg s.c.) or vehicle 20 min before the training phase. During the training phase, two identical objects were placed in the box and the animals were allowed to explore the objects for 10 min. Twenty-four hours later, one of the training objects was replaced with a new object of the same color but different shape, and the animals were again allowed to explore the objects for 10 min. Trials were video recorded and then the time spent exploring each object was scored by an experimenter blind to the group designation. The percent time spent exploring the novel object relative to the time spent exploring the familiar object was used as an indicator of recognition memory.

### Morris water maze

Brain-injured mice were trained to find the location of a hidden platform (12 cm in diameter) in a circular galvanized steel pool (1.2 m in diameter) located in a room with several extra maze cues mounted on the walls surrounding the pool. The pool was filled with water (maintained throughout training and testing at 20–22˚C) to a depth that reached 2 cm above the platform surface and made opaque with white non-toxic paint. Twenty minutes before the first trial on each training day, animals were s.c. administered either IGF-2 (30 μg/kg s.c.) or vehicle. For each training trial, a mouse was placed in the water maze at one of four randomly chosen release points facing the wall of the tank. The animal was then allowed to search for the hidden platform for 60 sec. If the mouse failed to find the hidden platform on any given trial, it was gently led there by the experimenter. Each mouse was allowed to remain on the platform for 30 sec before being removed and placed in a warming cage to await its next trial. Mice received four consecutive training trials with an intertrial interval of 4 min each day for 4 days, for a total of 16 training trials.

Twenty-four hours after the last training trial, the platform was removed and animals were given a 60-sec probe trial to test for spatial memory of the platform location. Latency to first crossing, number of crossings, and time spent in concentric circular regions centered on the previous platform location were used as measures of spatial memory.

### Statistical analyses

Data were analyzed using Sigma Plot 14. All data were examined for normality and equal variance before testing for statistical differences. Fear extinction was analyzed using a two-way repeated-measures analysis of variance (ANOVA). Contextual fear memory and NOR data were analyzed using two-tailed Student *t*-tests. Water maze training was evaluated using a two-way repeated-measures ANOVA. Spatial memory was statistically compared using either Student's *t-*test (i.e., latency to first platform crossing, number of crossings) or two-way repeated measures (i.e., dwell time in concentric circles centered on the platform), as appropriate. Bonferroni's *post hoc* tests were used to identify data points with significant differences between groups. Adjustments for multiple comparisons were made where appropriate. All data are reported as mean ± standard error of the mean (SEM), and *p* values were deemed statistically significant if <0.05.

## Results

### Pre-training insulin-like growth factor-2 enhances fear extinction in sham-operated, but not brain-injured, mice

**FIG. 1.**
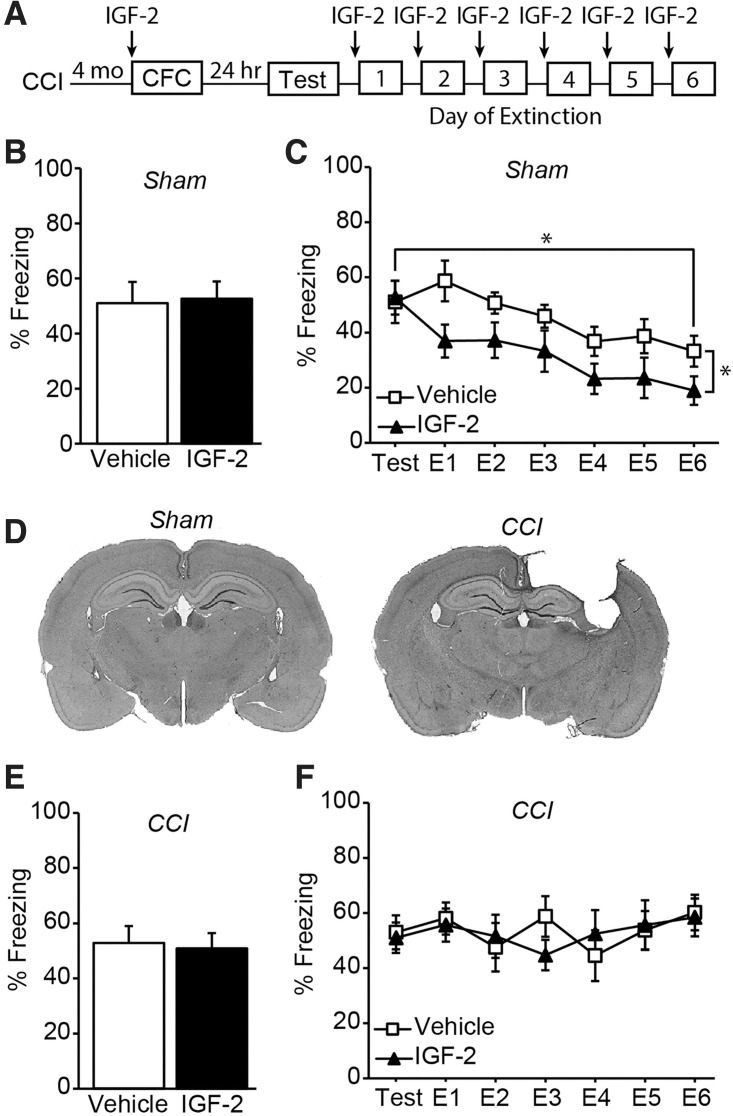
Pre-training IGF-2 enhances fear extinction in sham, but not CCI, mice. (**A**) Experimental timeline. Summary data (n = 7 per group) demonstrating that although (**B**) systemic IGF-2 administration does not improve fear memory tested at 24 h, it (**C**) facilitates the extinction of fear memory in sham-operated control mice. (**D**) Representative images of cresyl violet–stained sections from a sham and chronic CCI-injured animal. (**E**) Summary data (*n* = 7 per group) demonstrating that systemic IGF-2 administration does not improve fear memory in CCI-injured mice tested 4 months after injury. (**F**) In contrast to that observed in sham controls, systemic administration of IGF-2 to chronic CCI mice does not improve extinction of fear memory. Data are presented as the mean ± SEM. *p < 0.05. CCI, controlled cortical impact; CFC, contextual fear conditioning; IGF-2, insulin-like growth factor-2; SEM, standard error of the mean.

As a positive control for treatment efficacy, we initially tested whether 30 μg/kg of IGF-2 (s.c.) could enhance context fear memory or fear memory extinction in uninjured sham animals, as has been demonstrated in previous studies.^13–15,19,24^ A timeline of the sham or injury surgery, drug administration, and behavioral testing is outlined in Figure 1A. Before delivery of the mild foot shock on the first day of testing, all the sham animals were mobile and showed minimal freezing behavior in the training context (data not shown). Pre-training administration of IGF-2 to uninjured sham mice had no significant effect on fear memory when tested 24 h after training when compared to sham animals that received vehicle (*t*_(12)_ = 0.162, *p* = 0.874; Fig. 1B). However, over the 6 days of extinction training, systemic administration of 30 μg/kg of IGF-2 to sham animals facilitated extinction of fear memory, consistent with a previous report^15^ (two-way repeated-measures ANOVA, *F*_(1,12)_ = 6.318, *p* = 0.027; Fig. 1C).

We then questioned whether IGF-2 treatment would provide a similar cognitive enhancing effect in mice that had received a moderate-severe CCI injury 4 months earlier. Figure 1D shows representative cresyl violet–stained images from a sham animal and CCI-injured animal showing the magnitude of the injury. Cortical tissue loss and hippocampal shrinkage were observed ipsilateral to the injury, consistent with previous studies using moderate-severe CCI in mice.^25^ Figure 1E shows that pre-training IGF-2 administration had no effect on contextual fear memory, given that both the IGF-2- and vehicle-treated injured groups showed similar freezing rates during testing 24 h later (*t*_(12)_ = 0.243, *p* = 0.812). Unlike the enhancement of extinction observed in uninjured controls, systemic administration of 30 μg/kg of IGF-2 had no significant effect on fear extinction after moderate-severe CCI injury. There was no significant difference in freezing rates during extinction training trials between IGF-2-treated CCI animals and vehicle-treated CCI animals (*F*_(1,12)_ = 0.0087, *p* = 0.927; Fig. 1F). Further, there was no evidence of extinction memory over the course of training.

### Pre-training insulin-like growth factor-2 administration does not improve novel object recognition memory in controlled cortical impact–injured mice

**FIG. 2.**
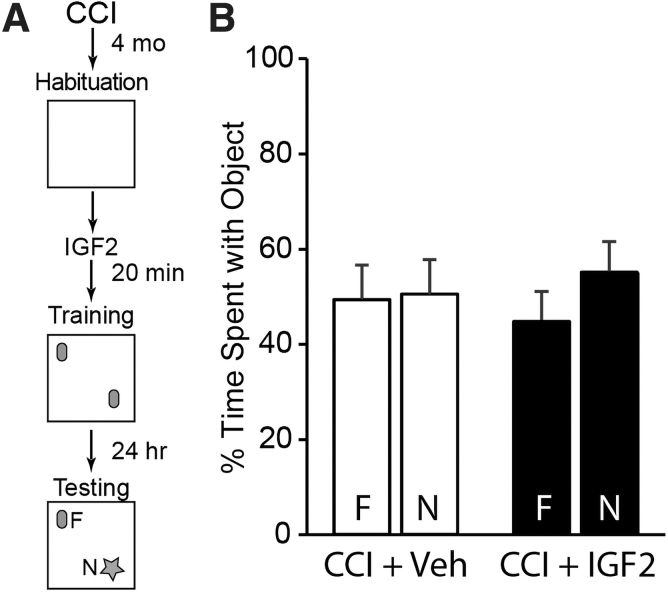
Systemic IGF-2 administration does not improve object recognition memory in injured mice. (**A**) Experimental timeline. (**B**) Summary data (*n* = 7 per group) from the testing session showing that both vehicle- and IGF-2-treated, CCI-injured mice spent equivalent time exploring the familiar (F) and novel (N) objects, indicating that IGF-2 treatment had no effect on reversing injury-induced object recognition memory deficits. Data are presented as the mean ± SEM. CCI, controlled cortical impact; IGF-2, insulin-like growth factor-2; SEM, standard error of the mean.

Given that IGF-2 administration failed to improve memory extinction in CCI mice, we questioned whether improved memory performance might be evident in other cognitive tasks previously shown in normal mice to be enhanced by IGF-2.^13–15,20,22,23^ TBI mice were s.c. injected with either 30 μg/kg of IGF-2 or vehicle as above, then trained and tested using a two-object NOR procedure as outlined in Figure 2A. During training, neither group showed an object or positional preference as evidenced by equivalent exploration times (vehicle: *t*_(12)_ = −0.556, *p* = 0.588; IGF-2: *t*_(12)_ = −0.68, *p* = 0.510; data not shown). Further, IGF-2 administration had no effect on long-term recognition memory in CCI animals assessed 24 h later, given that neither vehicle- (*t*_(12)_ = −0.108, *p* = 0.916) nor IGF-2-treated (*t*_(12)_ = −1.159, *p* = 0.269) groups showed a preference for attending to the novel object (Fig. 2B). In contrast, uninjured sham animals trained in the same task showed a significant preference for the novel object (*t*_(12)_ = −12.090, *p* < 0.001; Supplementary Fig. S1A).

### Spatial memory in controlled cortical impact–injured mice is not enhanced by pre-training insulin-like growth factor-2 administration

**FIG. 3.**
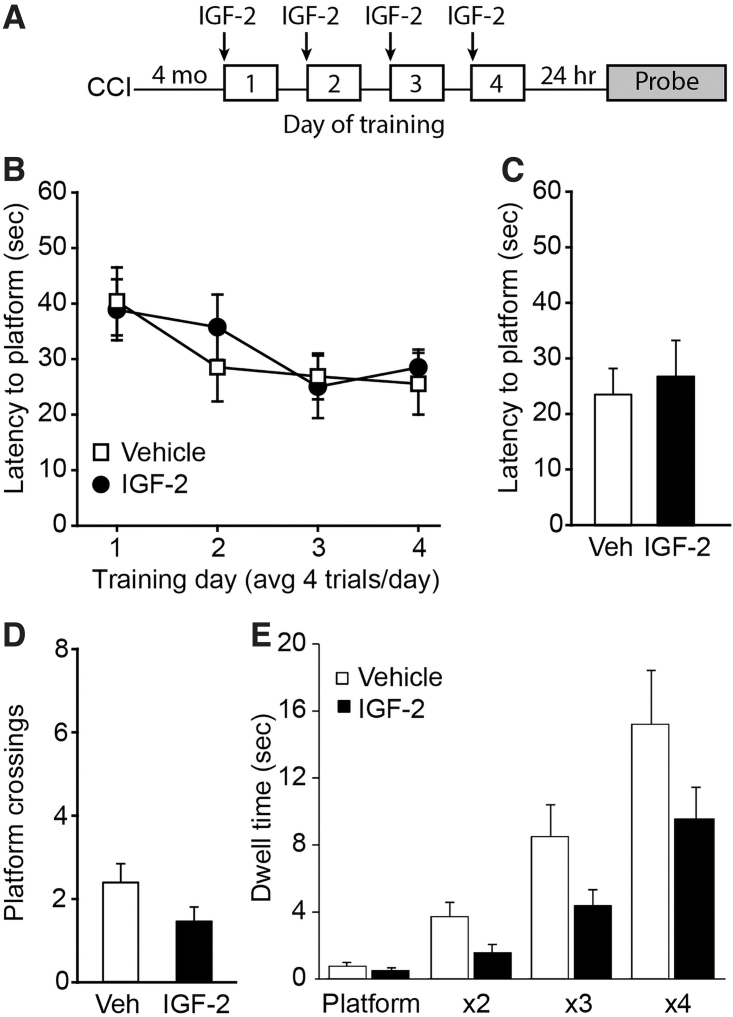
Spatial memory in CCI-injured mice was not enhanced by pre-training IGF-2 administration. (**A**) Experimental timeline. (**B**) Summary data (*n* = 7 per group) showing that there was no significant difference in acquisition of the hidden platform location between vehicle- and IGF-2-treated injured mice during training. When tested for their long-term memory, there were no significant differences detected between vehicle- and IGF-2-treated CCI mice in either (**C**) latency to the first platform crossing, (**D**) the number of platform crossings, or (**E**) time spent in concentric circles centered on the platform location. All data are presented as the mean ± SEM. CCI, controlled cortical impact; IGF-2, insulin-like growth factor-2; SEM, standard error of the mean; Veh, vehicle.

To determine whether IGF-2 could improve spatial memory in the chronic stage after TBI, CCI-injured mice were given IGF-2 (30 μg/kg s.c.) or vehicle 20 min before initiating each day's training in the MWM task (Fig. 3A). Although both the vehicle- and IGF-2-treated injured groups showed slightly improved latency to the platform over training days, there was no significant difference between the treatment groups (*F*_(1,11)_ = 0.0948, *p* = 0.764; Fig. 3B). When long-term memory was assessed, there were no significant differences detected between IGF-2- and vehicle-treated, brain-injured animals in their latency to the first platform crossing (*t_(12)_* = −0.410, *p* = 0.689; Fig. 3C), number of platform crossings (*t_(12)_* = 1.650, *p* = 0.125; Fig. 3D), or time spent in concentric circles centered around the former platform location (*F*_(1,12)_ = 1.538, *p* = 0.239; Fig. 3E), indicating that no benefit was derived from the IGF-2 treatment.

To demonstrate normal performance in the MWM task, a separate group of sham animals underwent similar training (Supplementary Fig. S1B–D). The training curve for uninjured sham animals was qualitatively similar to that of the chronic CCI groups. However, sham animals exhibited shorter latency to first platform crossing (sham: 11.62 ± 5.68 sec; CCI/vehicle: 24.09 ± 12.66 sec; CCI/IGF-2: 27.43 ± 17.44 sec), and more platform crossings (sham: 4.75 ± 2.50; CCI/vehicle: 2.57 ± 1.27; CCI/IGF-2: 1.57 ± 0.98 sec), indicating that sham uninjured animals could remember the location of the escape platform, whereas the vehicle- and IGF-2-treated injured animals could not.

## Discussion

IGF-2 has been identified as an important regulator of memory consolidation and enhancement, and exogenous administration of IGF-2 can improve memory performance in several animal models of disease.^5,13–15,19– 23,26,27^ To test the efficacy of pre-training administration of IGF-2 to improve memory in the chronic stage of TBI, animals were tested using multiple behavioral assessments 4 months after a moderate-severe CCI injury: fear memory extinction (Fig. 1), NOR (Fig. 2), and the MWM (Fig. 3). Our data showed that IGF-2 administration had no significant beneficial effect on memory performance in CCI-injured mice in any of these tasks. IGF-2 signaling is mediated through its receptor, IGF-2 receptor (IGF-2R; also known as cation-independent mannose-6-phosphate receptor).^11,12^ IGF-2R is expressed in the adult hippocampus and cortex,^7,28–31^ and a role for this receptor in memory enhancement has been recently reported.^27^

Consistent with this, s.c. administration of IGF-2 increased hippocampal expression of the plasticity-related genes, activity-regulated cytoskeleton-associated protein (Arc), c-Fos, and zinc finger-containing transcription factor 268 (Zif268).^13–15,27^ At present, it is not known whether the failure of IGF-2 to enhance memory in TBI mice may be related to altered expression/activity of IGF-2R or other components in its signaling pathway. Expression of plasticity-related genes (e.g., Arc, Zif268, and c-Fos) could be monitored to assess the intactness of the IGF-2R signaling pathway in the injured brain in the chronic stage of CCI.

In addition to neurons, IGF-2 receptors are located on capillaries, where they are thought to participate in receptor-mediated transport of IGF-2 across the blood–brain barrier.^32^ We observed enhanced context fear memory extinction after s.c. administration of IGF-2 to uninjured sham animals, suggesting that there was sufficient brain penetration. However, given that we did not measure whether hippocampal concentration of IGF-2 after systemic administration was comparable between sham and CCI mice, we cannot rule out the possibility that systemic IGF-2 administration to CCI mice did not reach a therapeutic concentration in the injured brain. Additional experiments would be needed to address whether a higher dose, or a different route of administration, of IGF-2 could enhance memory in the chronic stage of injury in CCI mice. Taken together, our results demonstrate that s.c. IGF-2 (30 μg/kg), which improved memory in normal mice, was insufficient to enhance cognitive performance in chronically brain-injured animals.

## Conclusion

A series of studies has provided experimental evidence that IGF-2 is a cognitive enhancer. These studies reported that IGF-2, when directly injected into brain structures or given systemically (s.c.), can enhance memory performance in normal animals.^13–15^ Further, IGF-2 attenuated age-related memory decline and improved cognitive function in a rodent model of autism spectrum disorder.^22,23^ Although we also observed that IGF-2 facilitated fear memory extinction in uninjured sham animals, our data indicated that IGF-2 was not effective at improving memory performance in CCI-injured mice when administered 4 months post-injury.

## Supplementary Material

Supplemental data

## Abbreviations Used

ANOVA: analysis of variance
Arc: activity-regulated cytoskeleton-associated protein
BSA: bovine serum albumin
CCI: controlled cortical impact
CFC: contextual fear conditioning
IGF-1: insulin-like growth factor-1
IGF-2: insulin-like growth factor-2
IGF-2R: IGF-2 receptor
MWM: Morris water maze
NOR: novel object recognition
PBS: phosphate-buffered saline
s.c.: subcutaneously
SEM: standard error of the mean
TBI: traumatic brain injury
Zif268: zinc finger-containing transcription factor 268
