# Supplementary material for: Insulin-Like Growth Factor-2 (IGF-2) Does Not Improve Memory in the Chronic Stage of Traumatic Brain Injury in Rodents
Source: Neurotrauma Rep. 2021 Oct 20;2(1):453–60. doi: 10.1089/neur.2021.0031 (PMC8655797; doi:10.1089/neur.2021.0031)
Supplement: Supplemental data [file Suppl_FigureS1.docx]

**

**

**Supplementary Figure 1. Performance of uninjured sham mice in the object recognition and spatial memory tasks.** (A) Uninjured, sham animals (n=7) were trained in the novel object task as outlined in the Methods section. Group data for the testing session show that uninjured animals spend significantly more time exploring the novel (N) object compared to the familiar (F) object, indicating intact recognition memory. (B) Spaced Morris water maze training curve for uninjured animals (n=7) showing an incremental improvement in the latency to platform over days of training. Twenty-four hours after the last training trial, animals were given a long term memory probe with the hidden platform removed from the arena, and the (C) latency to the previous platform location, and (D) number of platform location crossings were recorded. In contrast to the injured animals’ performance (shown in Figure 3), uninjured animals quickly swam to the location of the escape platform and crossed the previous location of the platform multiple times as they searched, indicating they retained a memory for the platform location. Data are presented as mean ± SEM. *, P<0.01.
